# SDN2GO: An Integrated Deep Learning Model for Protein Function Prediction

**DOI:** 10.3389/fbioe.2020.00391

**Published:** 2020-04-29

**Authors:** Yideng Cai, Jiacheng Wang, Lei Deng

**Affiliations:** ^1^School of Computer Science and Engineering, Central South University, Changsha, China; ^2^School of Software, Xinjiang University, Urumqi, China

**Keywords:** protein function, word embedding, convolutional neural network, deep multi-label classification, deep learning

## Abstract

The assignment of function to proteins at a large scale is essential for understanding the molecular mechanism of life. However, only a very small percentage of the more than 179 million proteins in UniProtKB have Gene Ontology (GO) annotations supported by experimental evidence. In this paper, we proposed an integrated deep-learning-based classification model, named SDN2GO, to predict protein functions. SDN2GO applies convolutional neural networks to learn and extract features from sequences, protein domains, and known PPI networks, and then utilizes a weight classifier to integrate these features and achieve accurate predictions of GO terms. We constructed the training set and the independent test set according to the time-delayed principle of the Critical Assessment of Function Annotation (CAFA) and compared it with two highly competitive methods and the classic BLAST method on the independent test set. The results show that our method outperforms others on each sub-ontology of GO. We also investigated the performance of using protein domain information. We learned from the Natural Language Processing (NLP) to process domain information and pre-trained a deep learning sub-model to extract the comprehensive features of domains. The experimental results demonstrate that the domain features we obtained are much improved the performance of our model. Our deep learning models together with the data pre-processing scripts are publicly available as an open source software at https://github.com/Charrick/SDN2GO.

## 1. Introduction

As an essential structural molecule, protein is a vital component of all biological tissues and cells and is also the primary bearer of life activities (Weaver, 2011). Understanding protein function is important both for biology and medicine and pharmacy. For example, clarifying the function of a protein can provide a target for genetic manipulation, and provide a reliable basis for designing a new protein or transform an existing protein, etc. So that, accurate annotation of protein functions is a significant and crucial task. Traditional experimental methods require a lot of resources and time to determine protein function, despite there are high accuracy and reliability. With the continuous development of high-throughput sequencing technology and genomics, the sequence of proteins has been exploded, but just a small percentage of the total known and predicted protein sequences have been extensively annotated regarding their functions. Currently, only <0.1% of the more than 179 million proteins in UniProtKB have been experimentally annotated (Consortium, 2019). However, it isn't straightforward to scale up the experimental method to accommodate such a large amount of protein sequence data, which urgently requires the development of computational methods to assist to annotate protein functions (Radivojac et al., 2013).

Gene Ontology, launched in 1998, is widely used in the field of Bioinformatics, and the original intention of GO was to provide a representative platform for terminology description or interpretation of words of genes and gene product characteristics. It enables Bioinformatics researchers to summarize, process, interpret, and share the data of genes and gene products (Ashburner et al., 2000). Gene Ontology is a Directed Acyclic Graph (DAG) type ontology. At present, GO contains more than 45,000 biological concepts include functions and cell locations, and is divided into three categories, covering three aspects of biology: Biological Process, Molecular Function, and Cellular Component. A protein generally has multiple GO annotations; therefore, protein function prediction is a very large-scale multi-label classification problem (Zhang and Zhou, 2013), and accurately assigning GO terms to proteins is a challenging task.

In recent years, some organizations and teams have developed algorithms, tools, and systems for protein function prediction using advanced computer technologies, such as machine learning and deep neural networks (Kulmanov et al., 2018; You et al., 2018, 2019; Hakala et al., 2019; Lv et al., 2019b; Piovesan and Tosatto, 2019; Rifaioglu et al., 2019; Kulmanov and Hoehndorf, 2020). Researchers predict protein functions from one or more of the followings: protein sequences (Kulmanov et al., 2018; You et al., 2018, 2019; Hakala et al., 2019; Piovesan and Tosatto, 2019; Kulmanov and Hoehndorf, 2020), protein structures (Yang et al., 2015; Zhang et al., 2018), protein protein interactions (PPI) network (Kulmanov et al., 2018; Zhang et al., 2018; You et al., 2019), and others (Kahanda and Ben-Hur, 2017; Hakala et al., 2019; Piovesan and Tosatto, 2019; Rifaioglu et al., 2019). For example specifically, GOLabeler (You et al., 2018) integrated five different types of sequence-based information and learned from the idea of web page ranking to train an LTR (learning to rank) regression model to receive these five types of information to achieve accurate annotation of GO terms. As a result, this model got the best overall performance among all submissions of the 3rd Critical Assessment of Function Annotation (CAFA3). NetGO (You et al., 2019), proposed by the GOLabeler team, is based on GOLabeler and incorporates massive amounts of protein-protein interaction (PPI) network information into the LTR framework. Compared with GOLabler, it has achieved a significant improvement in protein function prediction performance. Hakala et al. (2019) developed an integrated system, which obtain features from several different tools or methods: BLASTP, InterproScan, NCBI Taxonomy, NucPred, NetAcet, PredGPI, and Amino Acid Index (Kawashima and Kanehisa, 2000; Heddad et al., 2004; Kiemer et al., 2005; Pierleoni et al., 2008; Camacho et al., 2009; Federhen, 2012; Jones et al., 2014), and then respectively feed all the features to two classifiers based on neural network and random forest and finally combined the NN classifier and the RF classifier to achieve the best prediction performance. DeepGO (Kulmanov et al., 2018) encodes the amino acid sequence of the protein by trigrams and maps the trigrams to vector by one-hot encoding and dense embedding, and then feed it to a convolutional neural network (CNN) to extract the feature map. Next, a combined feature vector consisting of CNN features and PPI Network embedding features entered into the hierarchically structured classification layers for classification of GO terms. INGA2.0 (Piovesan and Tosatto, 2019) uses four components, Homology which inferred from sequence similarity, Domain architecture, protein-protein interaction networks, and integrated information from the “dark proteome” which include disordered and transmembrane regions, to predict protein function. This method has better capabilities to predict some extremely rare GO terms compared with others. Overall, these highly competitive models and systems have proven their outstanding performance in protein function prediction and are continually being optimized.

The amino acid sequence is crucial for understanding and analyzing proteins of various species. Some studies have shown that sequence homology-based BLAST methods are highly competitive in protein function prediction (Altshul, 1997; Gillis and Pavlidis, 2013; Hamp et al., 2013). Besides, there are several high-level physiological functions, such as apoptosis or rhythm regulation, which are often the result of the interaction of multiple proteins (Kulmanov et al., 2018), and according to the so-called “guilt-by-association” principle, interacting proteins should have some similar functions (Oliver, 2000; Schwikowski et al., 2000). Those shows that protein sequence information and PPI network information are essential to predict protein function. We have also noticed the critical position of the protein domain in protein-related features. The domain is a structural motif that exists independently in different combinations, and orders in the protein (Forslund and Sonnhammer, 2008) and is a higher-level protein component than the amino acid sequence (Richardson, 1981). Therefore, it makes sense to analyze and examine the effect of Domain content on protein function and try to use it to predict protein function. Besides, Machine Learning (ML) is currently popular and efficient for bioinformatics problems (You et al., 2018, 2019; Lai et al., 2019; Tan et al., 2019; Wang et al., 2019a; Zhu et al., 2019; Dao et al., 2020), especially, due to its strong ability to fit high-dimensional, sparse, and highly collinear complex data, deep learning technology has been widely used in bioinformatics fields, such as protein structure and function (Sønderby and Winther, 2014; Spencer et al., 2014; Wei et al., 2018; Kulmanov and Hoehndorf, 2020), gene expression regulation (Chen et al., 2016; Lanchantin et al., 2016), protein classification (Asgari and Mofrad, 2015; Sønderby et al., 2015), and structure and functions of nucleic acid (Zhang et al., 2016; Lv et al., 2019a; Wang et al., 2019a,b). For these considerations, here we proposed an integrated deep learning model based on protein sequences, protein domain content, and known protein-protein interaction networks to predict protein function. We first built three different neural network modules to learn features from protein sequences, domain content, and PPI Net separately, and then combined the features from these three different sources and inputted them to the neural network classifier to predict the probability of each GO term. The experimental results show that our method of adding domain content to predict protein function is successful, and our model achieved better performance than BLAST and two other recent high-performance methods on an independent dataset constructed using time-delay rules.

## 2. Materials and Methods

### 2.1. Data Source

#### 2.1.1. Training Data

- Sequence Data
  For our experiments, we downloaded the sequence information of the proteins needed for the research from the UniProt database as FASTA-format files (http://www.uniprot.org/downloads) (Consortium, 2015). Then a CD-hit tool was used to de-redundant the downloaded protein sequence data. We grouped proteins with a sequence similarity >60% into one cluster, and only one protein per cluster was retained. Finally, we obtained a benchmark for humans contains 13,704 proteins, and a benchmark for Yeast contains 6,623 proteins.
- Annotation Data
  We downloaded GO annotation data for proteins from GOA (http://www.ebi.ac.uk/GOA) (Barrell et al., 2009) published in December 2013. Please note that the GO annotation data here is for training only, and all data are annotated in 2013 or earlier. Finally, the annotation data contains 13,882 categories (9,221 in BP, 3,483 in MF, and 1,178 in CC) for Human and 4,796 categories (2,439 in BP, 1,733 in MF, and 624 in CC) for Yeast.
- Protein-Protein interaction (PPI) Network Data
  We have added protein-protein interaction (PPI) network data, which is derived from the STRING database v10 (https://string-db.org/) (Szklarczyk et al., 2015), to improve the performance of the experiment. Among them, human PPI data contains 11,759,455 scored links of 19,257 proteins, and Yeast's PPI data contains 1,845,966 scored links of 6,507 proteins.
- Protein Domain Data
  We downloaded protein domain data from the public database interpro (Hunter et al., 2009) (http://www.ebi.ac.uk/interpro/download/), which contains the all UniProtKB proteins and the InterPro entries and individual signatures they match. For a specific protein, we can obtain the types, quantity, and locations of all the domains it contains, and the start and the end positions in the protein sequence of a domain are indicated. We searched by the protein's UniProt ID to obtain the domain data of all the proteins we needed. Next, we performed de-redundancy; for the same domain information supported by contradictory evidence, we kept only one of them. In the end, our domain data contains 113,972 pieces of information of 14,242 domains for Human, and 23,326 pieces of information of 6,707 domains for Yeast.

#### 2.1.2. Independent Testing Data

The independent test data set is used for comparison with the competing methods. The collection of data generally follows the time-delayed rule of the CAFA challenge. We downloaded GO annotation data for proteins from GOA published in January 2016 and then obtained protein GO annotations added after 2013 (2014 and 2015). Specifically, we removed the annotation data published in December 2013 from the annotation data published in January 2016 and only retained the newly added protein annotation data. Next, we constructed an independent test benchmark based on the newly added annotation data; please note that all proteins contained in this benchmark do not have any GO annotations before 2014. Similarly, we filtered those proteins that were only annotated by GO terms that are extremely infrequent. The filtered independent test set contains 68 proteins for BP, 136 proteins for MF, and 106 proteins for CC.

### 2.2. Data Representation

#### 2.2.1. Protein Sequence Data

Protein sequence information is one of the inputs to our model. The sequence of each protein is a string composed of 20 specific amino acid codes with different lengths. In this experiment, we only selected proteins with a sequence length not exceeding 1,500. If the sequence length is <1,500, we padded zero at the end of the sequence to ensure that the length of each input protein sequence information is fixed. To fully extract the context and semantic knowledge of the sequence, we utilized the ProtVec of BioVec (Asgari and Mofrad, 2015), which is a biological sequence representation and feature extraction method, to map the sequence information. This method borrows the ideas of “word embedding” from Natural Language Processing (NLP) and obtains vector representations of biological sequences through training, and ProtVec is used for protein sequences. We followed ProtVec and used 3-grams encoding for protein sequences, that is, using a window of length 3 with a step size of 1 to slide the protein sequence to obtain a 3-grams sequence with a length of 1498 for each protein.

In order to convert 3-grams sequences information into vectors that can be received by the computing model, we used the ProtVec-100d-3grams table released by BioVec. We Downloaded this data from Harvard Dataverse (http://dx.doi.org/10.7910/DVN/JMFHTN). In this table, the protein vector is a distributed representation of proteins, and a 100-D vector presents each 3-gram. For our experiment, according to ProtVec, each protein will be represented as a 1,498 * 100 vector matrix, and then used as input to the model. In particular, according to the way we treat proteins <1,500 in length, if a 3-gram word contains one or more zeros we have padded, then the 3-gram will be represented as a 100D zero-vector.

#### 2.2.2. Protein Network Data

The protein network data we downloaded is scored links between proteins. The higher the score, the greater the probability of interactions between proteins. We filtered all scored links with 400 points, leaving only scored links whose score higher than 400, and then integrated the filtered protein network data into a PPI scored matrix. Each row of this matrix is a vector that represents the interaction of a protein with other proteins. If protein A interacts with another protein B in selected data, we set the value at the corresponding position in the vector to the fraction of these two proteins; otherwise, we set it to 0.

#### 2.2.3. Protein Domain Data

In proteins, the types and number of domains and the relative positions of different domains will affect the functions of the protein. To fully discover and extract the comprehensive information of the type, number, and position of domains in proteins to improve the performance of the model, we first need to sort the domains contained in each protein according to the information of positions in the domain data, so that we can obtain the information relative positions of different domains. However, the position information given by the database is only a possible range of domains in the protein sequence. For example, if the database provides the position of domain D in the sequence of protein P is 60–200, this only indicates that a domain D exists in the area of 60–200 in protein P, but we cannot obtain the actual length and location of this domain D. This is the result of technical limitations, which cause the existence of different domains to overlap, even a region completely contains another region, in a protein, and makes it challenging to sort domains.

In our experiments, we proposed a simple sorting method based on regional center points to solve this problem. Specifically, in a specific protein, there are three possibilities for the geographical relationship between any two different domains: detached, crossing, and containing. If the relationship is detached, we can quickly sort the two domains. If it is a cross-relationship or a containing-relationship, we calculated the center points of the two regions separately, and then put the domain with a forward center point in front of another one. After this, the information on the type, quantity, and relative position of the domain in the protein are obtained. Next, we learned from the idea of Natural Language Processing and treat each domain as a biological word, so the information of domains describing a specific protein is a biological sentence composed of some domain words in a particular order, while the functions of a protein are what the biological sentence means. The purpose of the domain module is to receive the biological sentence of protein and then abstract the features that represent the meaning of the sentence. Because the number of domains contained in different proteins is inconsistent, here we also need to solve the problem of the inconsistent size of model input. We obtained the maximum number of domains of proteins and used this maximum number (357 for Human and 41 for Yeast) as a standard and proteins with fewer domains than the maximum number were padded with 0. We encoded domains by word Embedding to input it into the model. Specifically, we utilized PyTorch's Sparse layer, which can initialize a simple lookup table to map sparse vectors to dense vectors, to generate a fixed lookup table for the domains. In this lookup table, each domain is represented by a 128-dimensional vector. In principle, the Sparse layer automatically maps high-dimensional one-hot vectors to low-dimensional dense vectors and provides the index of the dense vectors. The dimensions of both the one-hot vectors and the dense vectors are manually set by the user as needed, and we could get the required dense vector by entering the index. Therefore, the domains sentence of Human is represented by a 357*128 two-dimensional matrix, while the domains sentence of Yeast is represented by a 41*128 two-dimensional matrix. The Sparse layer will be integrated into the model and trained together, that is, as the model is continuously optimized, the representation vectors of domains in the lookup table will become increasingly accurate.

#### 2.2.4. Protein GO Terms

Given that a large number of specific GO terms often only exist in the annotation sets of a small number of proteins (You et al., 2018), and considering the calculation limit, we ranked the GO terms according to the number of annotations in proteins, and then use a set of thresholds (40 for BP, 20 for MF and 20 for CC) to select the GO terms, which contains 491 BP terms, 321 MF terms, and 240 CC terms, for Human, and a set of thresholds (10 for BP, 10 for MF and 10 for CC) to select the GO terms, which contains 373 BP terms, 171 MF terms, and 151 CC terms, for Yeast. We created three binary vectors for each protein to represent the labels of three sub-ontologies of GO: BP Ontology, MF Ontology, and CC Ontology. If a protein is annotated by a GO term, the value at the corresponding position of the label vector is set as 1, and otherwise is set as zero. Please note that all GO categories in the label vectors are selected.

### 2.3. Deep Model

**Figure 1:**
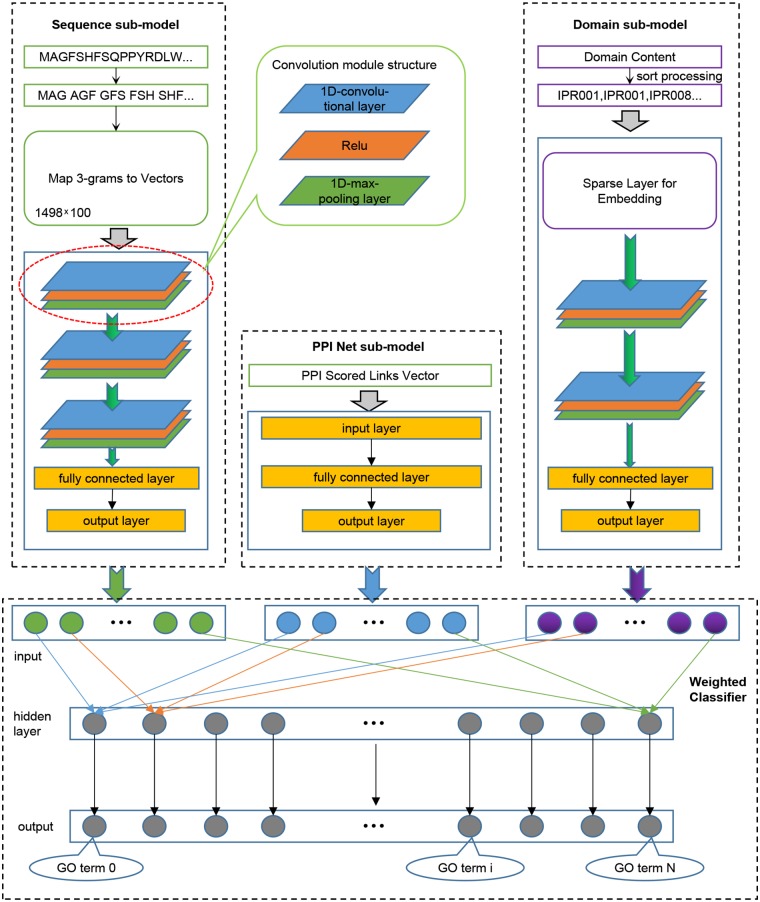
The integrated deep learning model architecture. (1) The Sequence sub-model utilizes 1-Dimensional convolutional neural networks to extract features from sequence input, which was encoded as 3-grams and then mapped to 3-grams-vector-matrix. (2) The PPI Net sub-model is generated to dense the features from PPI Network using classical neural networks. (3) The Domain sub-model initializes a Sparse layer, which is integrated into the sub-model to optimize, to generate a lookup table for domains, and the sorted domains sentence processed by the Sparse layer is entered into 1-Dimensional convolutional neural networks to extract features. (4) All the output features of the three sub-models are combined and entered into the Weighted Classifier, and the output vector represents the probability of GO terms.

We trained three models for the three sub-ontologies of GO. We randomly extracted 80% of the training data for iterative training of the model, and used the remaining 20% to verify the performance of the model after each iteration, and retained the model with the best generalization performance. Given that our model needs to receive input from three aspects of sequence, domain content, and PPI network information, as shown in Figure 1, we divided the model into four components: Sequence sub-model, Domain sub-model, PPI-Net sub-model, and Weighted Classifier.

#### 2.3.1. Sequence Sub-model

The input of this sub-model is a two-dimensional 3-grams-vector-matrix that represents protein sequence information. To extract in-depth high-dimensional features of protein biological sequences, we design and implement a model based on convolutional neural networks (CNN). The neural network is a mathematical algorithm model that mimics the behavioral characteristics of biological neural networks for distributed and parallel information processing (Haykin, 1994). In CNN, there is depth structure, and the input is convolved to obtain the output (LeCun et al., 1998), the convolution layer contains multiple convolution kernels, which can make the model extract more features in different aspects. In our experiment, we used a 1-Dimensional convolutional neural network, which uses a one-dimensional convolution kernel to perform convolution operations on the input data. After the sequence input is convolved to extract features, the output feature map is passed to the pooling layer for feature selection and information filtering; this is because the feature map still contains redundancy. Here, we use the max-pooling layer to treat the feature map. After processing, the selected feature map will be passed to the next layer as input. Specifically, three convolutional layers were set for the sequence sub-model, which were connected end to end. The feature map obtained after the convolution operation of each convolutional layer uses a maximum pooling layer to filter information to remove redundancy. The in-channels of the first convolutional layer are the same width as the input sequence information matrix and are set to 100. The in-channels of the other two convolutional layers are the same as the out-channels of the previous layer, and the out-channels of the three convolutional layers are set as 64, 32, and 16, respectively. For each convolution layer, a convolution kernel with a size of 16 is used for the convolution operation with a step size of 1. In order to completely extract the input features, padding was performed on the input with 0 before each convolution. Each maximum pooling layer is filtered using a kernel of size 2 with a step size of 2. The output feature map of the last pooling layer will be tiled into one dimension and input to the fully connected (FC) layers for dimensionality reduction. Finally, a feature vector representing the protein sequence information was obtained. The number of nodes in the output layer of the fully connected layer is set according to the number of three GO sub-ontology. Specifically, for Human, it was set as 491 for BP, 321 for MF, and 240 for CC, and for Yeast, it was set as 373 for BP, 171 for MF, and 151 for CC.

#### 2.3.2. PPI-Net Sub-model

In the PPI scored matrix, the feature vectors that characterize the interaction between proteins and other proteins have large dimensions, which are 18,901 for Human and 6,054 for Yeast, respectively, so we built a three-layer trapezoidal neural network module to dense the PPI features. In this module, the number of nodes in the input layer is the same as the dimension of the input feature vector, which is 18,901 for Human and 6,054 for Yeast. The number of nodes in the hidden layer is set to an intermediate value according to the number of nodes in the input layer and the output layer, which are 4,096 for Human and 2,048 for Yeast. And the size of the output layer is based on different species and GO sub-ontology, and is the same as the output layer of the Sequence sub-model.

#### 2.3.3. Domain Sub-model

The input of the Domain sub-model is the sorted protein domain content information. According to the input data, the first structure of the module is the integrated Sparse layer, the number of embedding is 14,243 for Human, and 6,708 for Yeast, and embedding dim are set as 128. For a specific protein, the output of the Sparse layer of the domain sentence input is a two-dimensional matrix. Therefore, similar to the sequence sub-model, we constructed a convolutional neural networks module containing two 1-D convolutional layers and two max-pooling layers. The in-channels of the first convolutional layer are set to 357 for Human, and 41 for Yeast, the in-channels of the second convolutional layer are consistent with the out-channels of the previous layer, and the out-channels of the two convolutional layers are set to 128 and 64. Besides, each convolutional layer used a convolution kernel of size 2 to perform a convolution operation with a step size of 2. In order to completely extract the input features, we padded the input with 0 before each convolution. The setting of the two maximum pooling layers is the same as the setting of the maximum pooling layer in the Sequence sub-model. The feature map output by the last pooling layer is tiled into one dimension and then input to the fully connected layers to reduce the dimension and the output layer of the fully connected layer. The size of the output layer is based on different species and GO sub-ontology, and is the same as the output layer of the Sequence sub-model.

#### 2.3.4. Weighted Classifier

**Figure 2:**
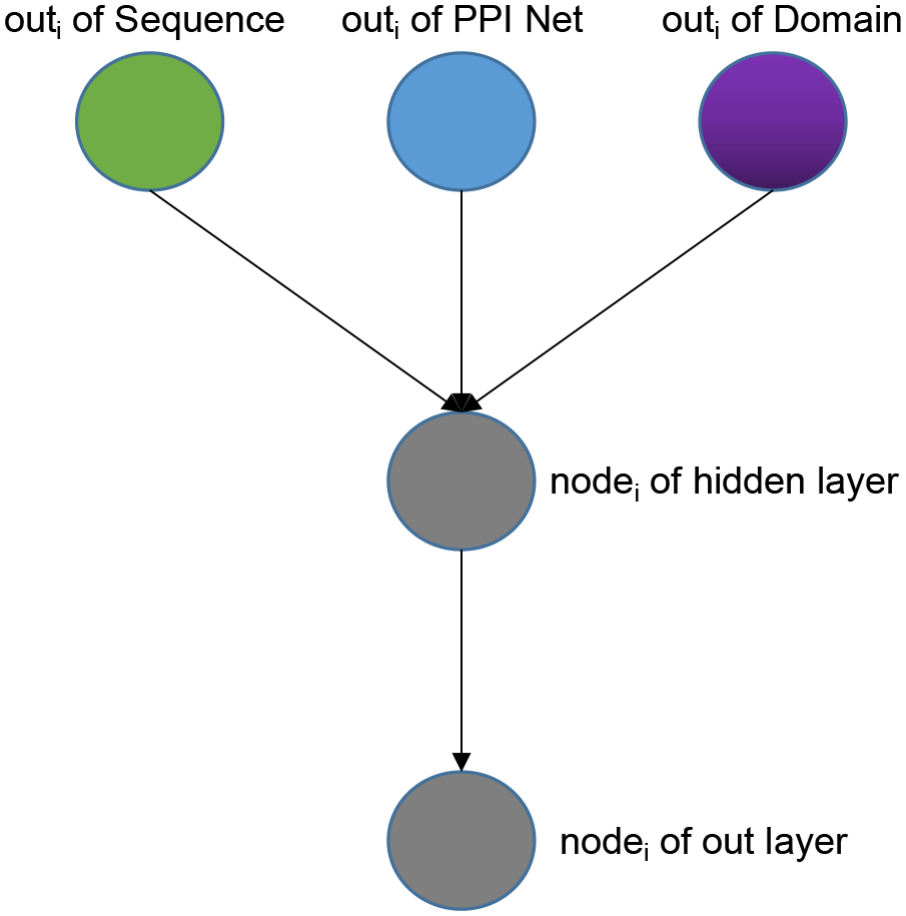
The architecture of one single GO classifier in the weighted classifier.

Weighted Classifier accepts output vectors from three sub-models: Sequence sub-model, Domain sub-model, PPI-Net sub-model. Through training, each GO classifier learns and optimal the weights that receive the features from three sub-models to achieve the best effect of multi-label classification. Note that the output vectors of the three modules have the same dimensions. As a whole, our Weight Classifier is a three-layer non-fully connected network model. The number of nodes in the input layer is the sum of the number of output nodes of the three sub-models, and both the nodes of hidden layer and the nodes of out layer are the same as nodes of the output layer of the three sub-models, which are set according to different species and GO sub-ontology. From the perspective of a single GO classifier, the structure is shown in Figure 2. For a specific GO classifier, the hidden node only accepts three features, which are from the corresponding position of the output vector of three sub-model, respectively, corresponding to the GO category, and to extract the corresponding area, we used a binary mask matrix to implement this connection control. The output node of the Classifier also only receives the output of the corresponding hidden node, and we also used a binary mask matrix to implement connection control. In general, let the entire Weight Classifier as a whole again, each node in the hidden layer is only connected to the three corresponding nodes in the output layer, and each node in the output layer is connected to only one corresponding hidden layer node. Therefore, the weights between the hidden layer nodes and the input layer nodes represent the preference of the Classifier for features from three sub-models, and the weights between the output layer nodes and hidden layer nodes globally balance the output values of the Classifier to the same level.

For all components of the model, we used the Rectified-linear-unit (ReLU) (Glorot et al., 2011), which could improve the computational efficiency and retain gradient (Nair and Hinton, 2010), as the activation function. Besides, by running specific optimization algorithms to minimize the loss function, the DNN model can be iteratively optimized by updating the weights and biases. Especially, the model is trained using an adaptive optimizer, Adam (Kingma and Ba, 2014).

### 2.4. Evaluate Methods

We evaluate the performance of the model through three measures, which are F-max, AUPR (area under the precision-recall curve), and AUC (area under the receiver operator characteristics curve), where F-max and AUC are used in the CAFA challenge (Radivojac et al., 2013). We use the standard provided by CAFA to calculate F-max and the formulas as follows:

(1) $$\begin{array}{r} F_{max}=\underset{t}{\max}\left\{\frac{2\cdot pr\left(t\right)\cdot rc\left(t\right)}{pr\left(t\right)+rc\left(t\right)}\right\} \end{array}$$

where *pr*(*t*) and *rc*(*t*), respectively represent precision and recall of the threshold *t* ∈ [0, 1], and can be calculated by the following formulas:

(2) $$\begin{array}{r} pr\left(t\right)=\frac{1}{m\left(t\right)}\cdot \overset{m\left(t\right)}{\underset{i=1}{\sum }}pr_{i}\left(t\right) \end{array}$$

and

(3) $$\begin{array}{r} rc\left(t\right)=\frac{1}{n}\cdot \overset{n}{\underset{i=1}{\sum }}rc_{i}\left(t\right) \end{array}$$

where *m*(*t*) is the number of proteins that annotated with at least one GO term using a threshold *t*, *n* is the total number of proteins in the target data set. *pr*_*i*_(*t*) and *rc*_*i*_(*t*) represent the precision and recall of a specific protein *i* using a threshold *t*, and are calculated by the following formulas:

(4) $$\begin{array}{r} pr_{i}\left(t\right)=\frac{\sum_{f}I\left(f\in P_{i}\left(t\right)\wedge f\in T_{i}\right)}{\sum_{f}I\left(f\in P_{i}\left(t\right)\right)} \end{array}$$

and

(5) $$\begin{array}{r} rc_{i}\left(t\right)=\frac{\sum_{f}I\left(f\in P_{i}\left(t\right)\wedge f\in T_{i}\right)}{\sum_{f}I\left(f\in T_{i}\right)} \end{array}$$

where *f* is a functional term in the ontology, Function *I*(·) is the standard indicator function. *T*_*i*_ is the set of true labels for protein *i*, and *P*_*i*_(*t*) is the set of predicted labels for protein *i* using a threshold *t*. Once the precision and recall that calculated by different values of t for a particular functional term were determined overall proteins, we could then calculate the AUPR using the trapezoid rule. Compared with AUC, AUPR has a greater penalty for false positives[6].

We also calculate the AUC value for each model of the GO sub-ontology, and the calculation formulas are as follows:

(6) $$\begin{array}{r} AUC=\int_{-\infty }^{\infty }TPR\left(t\right)\left(-FPR\left(t\right)\right)dt, \end{array}$$

(7) $$\begin{array}{r} TPR\left(t\right)=\frac{TP\left(t\right)}{TP\left(t\right)+FN\left(t\right)} \end{array}$$

and

(8) $$\begin{array}{r} FPR\left(t\right)=\frac{FP\left(t\right)}{FP\left(t\right)+TN\left(t\right)} \end{array}$$

where *TP* is the number of true positives, *FP* is the number of false positives, and *TN* is the number of true negatives, *FN* is the number of false negatives.

### 2.5. Model Implementation and Computing Environment

We used PyTorch, a Python-based deep learning framework, to implement our model. To speed up the training process, we used a *RHEL* server with four *NVIDIACorporationGM*107*GL* graphics cards installed and total video memory of 32 GB. Under a set of parameters, the whole training time for the most computationally-intensive BP model is <10 h. In terms of prediction, in the case where the sequence, domain, and PPI input information of the predicted protein has been processed in advance, using an optimized model to predict 1,000 proteins takes about 6 min.

## 3. Results

### 3.1. Experiment

Owing to the complexity of our model composition and the requirement to determine a large number of hyperparameters, we first pre-trained the three-component sub-models of Sequence, Domain, and PPI Net. We used the GO annotations of proteins as a label and calculated the binary cross-entropy between the predicted values and the actual values, and use this as the loss to back-propagate to update the weights and biases between the nodes connected in the model. We manually adjusted the hyper-parameters, such as the learning rate and batch-size of each module, and selected the optimal model based on the validation loss value using the training set. After adjusting the parameters of the three sub-modules, we used the output of these three fine-tuned models as input to manually adjusted the hyperparameters of the Weighted Classifier, and also select the optimal model based on the validation loss value using the training set. Tables S1–S4 shows the details of the training of different hyperparameters.

We used 5-fold cross-validation on the training set to test the performance of the model, and the results are shown in Table 1. It is clear that the model has achieved a favorable F-max value for each sub-ontology of GO, which indicates that our method is an effective protein function prediction method.

**Table 1:** The 5-fold cross validation results of training data.

**Method**	**BP**			**MF**			**CC**		
	***F*_max_**	**AUPR**	**AUC**	***F*_max_**	**AUPR**	**AUC**	***F*_max_**	**AUPR**	**AUC**
SN2GO (human)	0.473	0.441	0.908	0.546	0.527	0.938	0.587	0.600	0.949
SDN2GO (human)	**0.507**	**0.487**	**0.921**	**0.653**	**0.655**	**0.957**	**0.601**	**0.617**	**0.952**
SN2GO (yeast)	0.414	0.289	0.810	0.548	0.435	0.870	0.520	0.395	**0.881**
SDN2GO (yeast)	**0.415**	**0.304**	**0.839**	**0.611**	**0.530**	**0.903**	**0.528**	**0.424**	0.878

*The bold values indicate the best values*.

### 3.2. Evaluating the Performance of Using Domain Content

Using the comprehensive information of types, quantities, and positions of protein domain content for prediction of protein function is the crucial component and emphasis of this research. In order to explore and explain the critical role of comprehensive domain information on protein function prediction, the deep models without the domain module were constructed for three sub-ontology of GO, and each model contained only the Sequence sub-model, PPI-Net sub-model, and Weighted Classifier, and we named it SN2GO. Among SN2GO, since the Sequence sub-model and PPI-Net sub-model in the SDN2GO model are pre-trained separately, the structure and hyperparameter settings of the Sequence sub-model and the PPI-Net sub-model are the same as those of the corresponding modules in the SDN2GO model, and the Weighted Classifier removes the relevant part of the domain from the input layer, the settings of the hidden layer and output layer are still the same as those of the SDN2GO Weighted Classifier. To ensure fairness of comparison, we also manually readjusted the learning rate and batch size hyperparameters and selected the optimal Weighted Classifier model for SN2GO.

**Figure 3:**
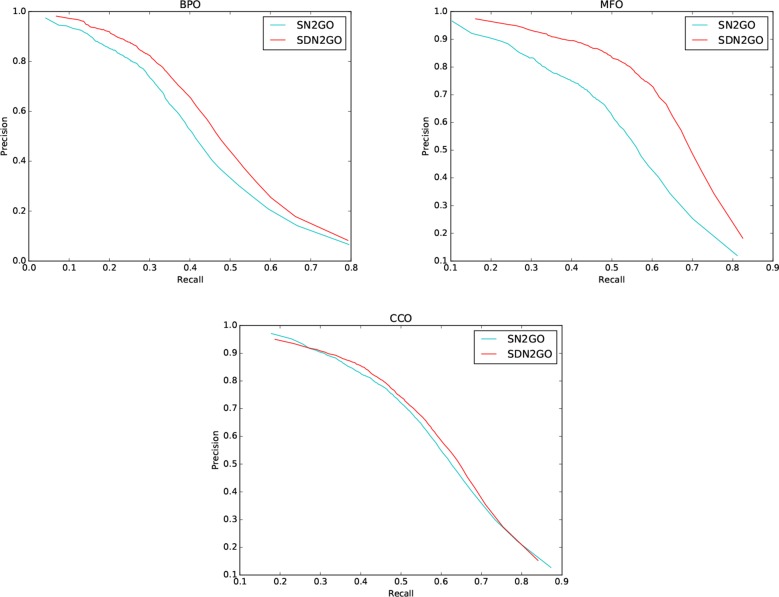
Precision-recall (P-R) curves of SDN2GO and SN2GO. The performances of the two methods were evaluated on the validation data of human in each sub-ontology of GO (gene ontology).

We observed the performance of SN2GO on the training set and compared it with SDN2GO. As the same, we used SN2GO to perform a 5-fold cross-validation experiment on the training set. Table 1 shows the cross-validation results of SN2GO. We find that compared with SN2GO, the performance of the SDN2GO that uses domain information has been significantly improved on all the sub-ontology of GO, especially in the MF Ontology of humans, the F-measure value of SDN2GO has been enhanced by nearly 20% (0.65 vs. 0.55) compared to SN2GO. As shown in Figure 3, the PR curves of SDN2GO and SN2GO on validation data of humans, it is clear that the red PR curve surrounds the other one on each sub-ontology. This result shows that domain information plays an essential role in protein function prediction, and proves that our coding and processing methods for protein domain information and the sub deep learning models for domains are useful and meaningful.

### 3.3. Comparison With Competing Methods

In order to further verify the performance of SDN2GO, we compared the two novel methods, NetGO and DeepGO, on the independent test set. Both of these two methods are competitive and excellent in protein function prediction and have achieved outstanding results on some datasets. As a state-of-the-art machine learning method for protein function prediction, NetGO provides constructive ideas on how to integrate features based on different sources. At the same time, DeepGO is quite representative of using deep learning technology for protein function prediction. Specifically, NetGO integrates five different types of sequence-based evidence and massive network information into the learning to rank (LTR) framework to predict protein function. We uploaded the protein sequence of the independent test set in Fasta format to the AFP (automated function prediction) webserver (http://issubmission.sjtu.edu.cn/netgo/) released by NetGO and then downloaded the prediction result of NetGO in txt format after a while. DeepGO uses convolutional neural networks to extract protein sequence features and combines known PPI network information as combined features to predict protein functions. We downloaded all source code of DeepGO from GitHub and downloaded the required data, and the fine turned neural network models saved in PKL format from the provided webserver (http://deepgo.bio2vec.net/data/deepgo/), and then entered the test protein sequence in Fasta format to this open-source tool, and obtained the prediction results of DeepGO. Besides, the BLAST was also used in comparative experiments.

**Figure 4:**
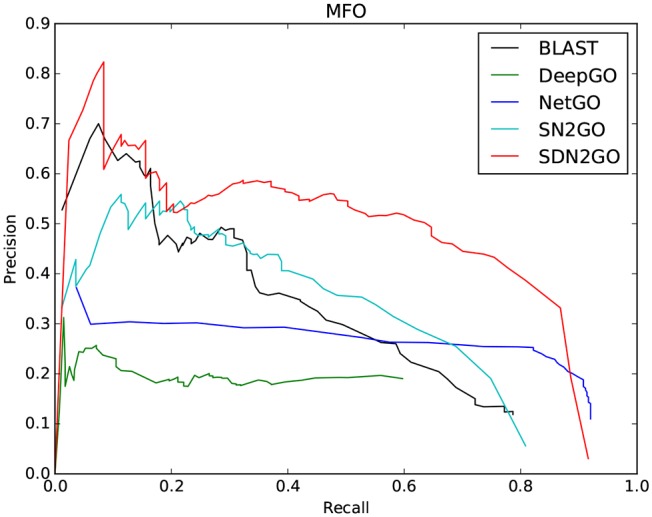
Precision-recall (P-R) curves of BLAST, DeepGO, NetGO, SN2GO, and SDN2GO. The performances of the five methods were evaluated on the independent testing set in MFO (molecular function ontology).

The comparison results are shown in Table 2. We have observed that BLAST performs well on every GO sub-ontology, which illustrates again that the sequence homology-based BLAST method is still quite competitive. NetGO and DeepGO performed well on MFO and BPO, respectively, but did not achieve their claimed effects on other sub-ontology. We further analyzed the prediction results of these two methods, and we found that the false-positive rates of both of them are relatively high, which leads to their inability to obtain high precision values. Figure 4, which shows the PR curves of MFO on independent test sets for various methods, demonstrates our analysis results from one aspect. The PR curves of BPO and CCO and other specific details can be seen in Figures S1, S2. Obviously, SDN2GO outperformed other methods on all sub-ontologies, especially on MFO. Those shows that our model has excellent generalization performance and is a currently competitive method for protein function prediction. In particular, we paid attention to the performance of SN2GO, which lacks the domain sub-model on the test set. The results show that its performance on BPO and MFO is far worse than that of SDN2GO, and prove that extracting features from protein domains for protein function prediction is feasible, and will improve the accuracy of GO term labeling for proteins, especially on BPO and MFO.

**Table 2:** The comparison results of the competing method on the independent testing set.

**Method**	**BP**			**MF**			**CC**		
	***F*_max_**	**AUPR**	**AUC**	***F*_max_**	**AUPR**	**AUC**	***F*_max_**	**AUPR**	**AUC**
BLAST	0.347	0.192	0.771	0.381	0.292	0.873	0.386	0.245	0.860
DeepGO	0.321	0.095	0.729	0.291	0.117	0.784	0.210	0.080	0.687
NetGO	0.173	0.048	0.594	0.386	0.243	0.919	0.217	0.092	0.669
SN2GO	0.132	0.044	0.893	0.423	0.306	0.953	0.384	0.264	**0.948**
SDN2GO	**0.361**	**0.203**	**0.917**	**0.561**	**0.471**	**0.964**	**0.432**	**0.290**	0.947

*The bold values indicate the best values*.

## 4. Discussion

SDN2GO, an integrated deep learning-based weight model we have proposed, combines three aspects of information: protein sequence, protein domain content, and known protein-protein interaction networks. We constructed three sub-models for these three aspects of information, and then learned and extracted three components of features through pre-training the sub-models. Each GO term of the protein was finally scored and annotated through the integrated deep learning weight classifier. The 5-fold cross-validation results show that SDN2GO is a stable and reliable method for protein function prediction. In order to further verify the generalization performance and competitiveness of SDN2GO, we constructed an independent test set based on the principle of time-delay for comparison with the novel method and the classic BLAST method. The comparison results show that our method has achieved the maximum F-max value for each sub-ontology of GO.

Many studies illustrated that protein sequence and PPI network are valid for protein function (Kirac and Ozsoyoglu, 2008; Jiang and McQuay, 2011; Nguyen et al., 2011; Baryshnikova, 2016; Kulmanov et al., 2018). Besides, some researchers have used protein domain information to predict protein function (Altshul, 1997; Forslund and Sonnhammer, 2008), but they only focused on a single aspect of type or structure of the domain and failed to fully mine the general characteristics of various aspects of the domain. We considered this and drowned lessons from the principle of NLP to encode domains to integrate the type, quantity, and position information of the protein domains, and utilized the convolutional neural network to extract the general characteristics of the domains, which is the advantage of our model. We built a comparison model SN2GO based on SDN2GO without domain sub-model and conducted comparative experiments on both the training data and the independent test set. The results show that the domain information has significantly improved the prediction effect of the model, especially in BPO On MFO; this might be because the domain information, as a higher-level protein feature than sequence, is more intuitive in expression and closer to the functions of the protein. And to a certain extent, the comparison results illustrated the correctness and generalizability of our methods of protein domain information processing and feature extraction.

In the future, we will continue to improve our model, such as adding more GO annotation categories to expand the scale of multi-label classification. Besides, we will also try to integrate more aspects of protein-related features, such as protein structure information and co-expression information, into our model to explore the role of different information on protein function prediction.

## Data Availability Statement

Publicly available datasets were analyzed in this study. This data can be found here: https://github.com/Charrick/SDN2GO/tree/master/data.

## Author Contributions

YC and LD conceived this work and designed the experiments. YC and JW built the experimental environment. YC carried out the experiments. YC, LD, and JW collected the data and analyzed the results. YC and LD wrote, revised, and approved the manuscript.

## Conflict of Interest

The authors declare that the research was conducted in the absence of any commercial or financial relationships that could be construed as a potential conflict of interest.
