# Supplementary material for: SDN2GO: An Integrated Deep Learning Model for Protein Function Prediction
Source: Front Bioeng Biotechnol. 2020 Apr 29;8:391. doi: 10.3389/fbioe.2020.00391 (PMC7201018; doi:10.3389/fbioe.2020.00391)
Supplement: Supplementary file 1 [file Image_1.pdf]

Supplementary Material

1 SUPPLEMENTARY TABLES AND FIGURES

1.1 Tables and Figures

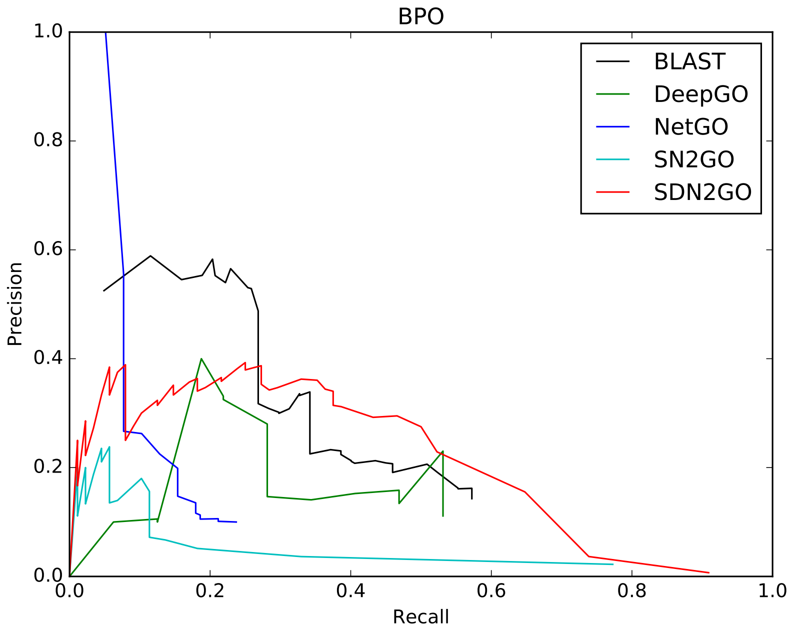

Figure S1. Precision-recall (P-R) curves of BLAST, DeepGO, NetGO, SN2GO, and SDN2GO. The performances of the five methods were evaluated on the independent testing set in BPO(Biological Process Ontology)

Table S1. Sequence sub-model hyperparameter adjustment results.

learning rate	batch size	convergence epoch	loss
0.01	8	17	0.0510
0.01	16	11	0.0482
0.01	32	28	0.0421
0.01	64	38	0.0443
0.01	128	40	0.0536
0.001	8	10	0.0362
0.001	16	10	0.0365
0.001	32	18	0.0395
0.001	64	20	0.0383
0.001	128	26	0.0407
0.0001	8	20	0.0376
0.0001	16	28	0.0416
0.0001	32	31	0.0419
0.0001	64	36	0.0383
0.0001	128	40	0.0403
0.00001	8	38	0.0408
0.00001	16	39	0.0417
0.00001	32	40	0.0442
0.00001	64	38	0.0482
0.00001	128	37	0.0477

Table S2. PPI Net sub-model hyperparameter adjustment results.

learning rate	batch size	convergence epoch	loss
0.01	8	55	1.0480
0.01	16	18	1.2462
0.01	32	1	0.3400
0.01	64	1	0.3220
0.01	128	2	0.3290
0.001	8	25	0.1040
0.001	16	26	0.0475
0.001	32	24	0.0398
0.001	64	29	0.0338
0.001	128	30	0.0344
0.0001	8	30	0.0335
0.0001	16	24	0.0342
0.0001	32	21	0.0350
0.0001	64	30	0.0340
0.0001	128	30	0.0326
0.00001	8	52	0.0335
0.00001	16	53	0.0327
0.00001	32	58	0.0317
0.00001	64	58	0.0362
0.00001	128	59	0.0405
0.000001	8	59	0.0404
0.000001	16	60	0.0432
0.000001	32	60	0.0505
0.000001	64	60	0.0710
0.000001	128	49	0.3440

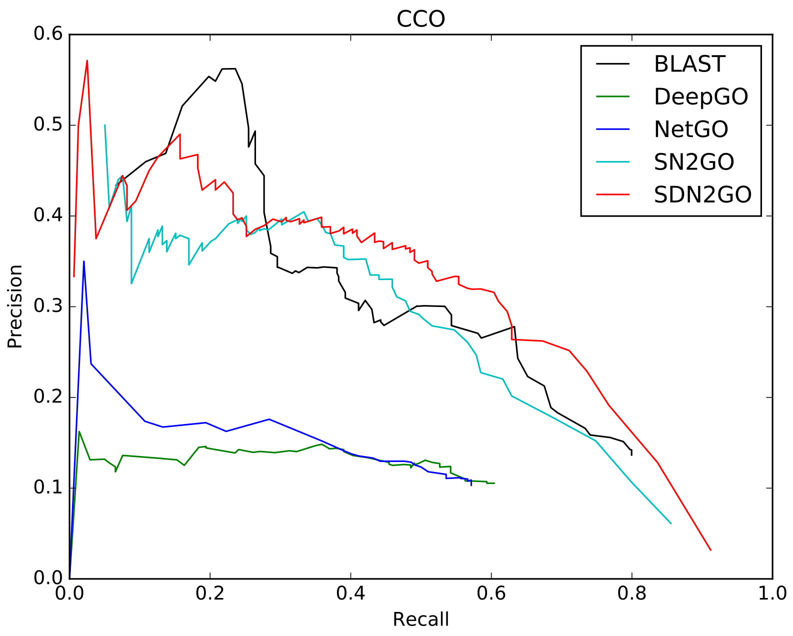

Figure S2. Precision-recall (P-R) curves of BLAST, DeepGO, NetGO, SN2GO, and SDN2GO. The performances of the five methods were evaluated on the independent testing set in CCO(Cellular Component Ontology)

Table S3. Domain sub-model hyperparameter adjustment results.

learning rate	batch size	convergence epoch	loss
0.01	8	97	0.0330
0.01	16	98	0.0292
0.01	32	100	0.0299
0.01	64	82	0.0286
0.01	128	60	0.0288
0.001	8	30	0.0267
0.001	16	21	0.0261
0.001	32	24	0.0267
0.001	64	37	0.0276
0.001	128	59	0.0297
0.0001	8	74	0.0290
0.0001	16	77	0.0279
0.0001	32	95	0.0286
0.0001	64	100	0.0277
0.0001	128	99	0.0332
0.00001	8	100	0.0316
0.00001	16	100	0.0378
0.00001	32	100	0.0431
0.00001	64	99	0.0431
0.00001	128	98	0.0472

Table S4. Weighted classifier hyperparameter adjustment results.

learning rate	batch size	convergence epoch	loss
0.01	8	23	0.0322
0.01	16	28	0.0314
0.01	32	29	0.0301
0.01	64	30	0.0302
0.001	8	54	0.0297
0.001	16	51	0.0289
0.001	32	51	0.0293
0.001	64	58	0.0293
0.0001	8	59	0.0300
0.0001	16	60	0.0305
0.0001	32	60	0.0325
0.0001	64	60	0.0378
